# *Agaricus bisporus* production on substrates pasteurized by self-heating

**DOI:** 10.1186/s13568-017-0438-6

**Published:** 2017-06-24

**Authors:** Stephania Colmenares-Cruz, José E. Sánchez, Javier Valle-Mora

**Affiliations:** 1 Instituto de Biociencias, Blvd. Príncipe Akishino S/N, Solidaridad 2000, 30798 Tapachula, Chiapas Mexico; 2 El Colegio de la Frontera Sur., Carretera al Antiguo Aeropuerto km 2.5, 30700 Tapachula, Chiapas Mexico

**Keywords:** Portobello, Substrate preparation, Button mushroom, Mushroom cultivation

## Abstract

The aim of this work was to determine if the self-heating pasteurization procedure is technically applicable to the cultivation of *Agaricus bisporus*. Firstly the substrates alone (corncob, Pangola grass and a mixture of both ingredients with wood shavings) were tested. Two supplementation trials were then undertaken using soybean, wheat bran, sheep manure, sesame seed, black bean and chia. Highest production values (BE = 176.3% and Y = 26.6 kg/m^2^) were obtained using a 9% supplement, with a formula consisting of 25% each of soybean, black bean, wheat bran and chia, added at spawning and at casing. These results were comparable to those obtained with the Phase II compost traditionally used for *A. bisporus* cultivation.

## Introduction

*Agaricus bisporus* is the fourth mushroom species cultivated in the world, with 15% of global production (34 × 10^6^ t edible mushrooms, Royse et al. 2016). The method usually used to cultivate the white button mushroom was described by Sinden and Hauser (1950) and improved through the years by a great number of research findings (Wuest 1982). The process involves two composting phases and is used worldwide because of excellent results. Phase I is a composting treatment requiring between 6 and 14 days, according to raw materials used. Phase I helps soften straw and other raw materials, break down soluble sugars and lower the C/N ratio. Biological (bacterial) and chemical (ammonia) activities increase during Phase I composting (Straatsma et al. 1995; Gerrits et al. 1995). This phase brings environmental challenges to growers because of odors and slurries (Mamiro et al. 2007; Beyer 2017). Phase II is a pasteurization process devoted to reduce competitor microbiota and give the substrate its biological selectivity. In Phase II, ammonia is reduced to levels that are non-toxic to *A. bisporus* (Laborde et al. 1993). Besides environmental problems, the Phase II technology for producing *A. bisporus* requires time, labor and investment (Miller et al. 1990). The loss of matter during composting is also an argument for the development of mushroom cultivation alternative methods.

*Agaricus bisporus* is considered a litter secondary decomposer, which means that bacteria and other fungi have to break down raw materials before the mushroom can grow, however Till (1962), demonstrated that *A. bisporus* could be cultivated on a non-composted substrate, like autoclaved sawdust. Since that time, several studies about cultivation (San Antonio 1971; Sanchez and Royse 2001; Bechara et al. 2005, etc.) and also on ligninolytic activity of this mushroom (Durrant et al. 1991; Wood and Leatham 1983) have placed *A. bisporus* as a basidiomycete able to degrade lignin and to grow on several raw, non-composted substrates.

Several alternative methods have been developed to grow *A. bisporus*. They consist in preparing a non-composted sterilized or pasteurized substrate at different temperatures, for avoiding the long composting traditional procedure (Till 1962; Mee 1978; San Antonio 1971; Sanchez and Royse 2001; Bechara et al. 2005, 2006; Coello-Castillo et al. 2009). However, none of them have been applied commercially.

A low input technology has been proposed to cultivate oyster mushrooms *Pleurotus ostreatus* (Villa-Cruz et al. 1999; Hernández et al. 2003; Barrios-Espinoza et al. 2009; Avendaño-Hernandez and Sánchez 2013). This technology is based upon the use of substrates pasteurized by self-heating. It consists on mixing raw materials with calcium hydroxide (2%) at controlled moisture levels and depositing the substrate in a wooden crate to allow heat to warm up the entire mass for pasteurization purposes. The entire process is accomplished within two days, and has been used successfully to cultivate strains of *Pleurotus* species like *P. djamor*, *P. citrinopileatus* and *P. eryngii,* and also other edible mushroom genera including *Auricularia* and *Agrocybe* (Morales and Sánchez 2017).

The aim of this work was to determine if the self-heating pasteurization procedure is technically applicable to *A. bisporus* cultivation, and if production yields are comparable to those obtained using the two-phase traditional method.

## Materials and methods

### Strains

For spawning, the commercial variety Portobello Heirloom of *Agaricus bisporus* was purchased from Amycel (San Miguel de Allende, Guanajuato, México). Inocula of one or five percent were used as indicated.

### Substrates and supplements

Three cultivation substrates were tested using the following agricultural byproducts obtained from local markets: (1) Pangola grass (*Digitaria decumbens*) cut into 2–2.5 cm lengths, (2) corncob (*Zea mays*) cut into 1–2 cm lengths, and (3) a mixture of wood shavings of the primavera tree (*Tabebuia rosea*) (16.6%), with corncob (41.6%) and Pangola grass (41.6%). These substrates were combined with 2% hydrated lime Ca(OH)_2_ (Super Cal hidratada Grijalva), and the moisture was adjusted to 65% with tap water.

The following supplements were obtained locally: soybean (*Glycine max*), wheat bran, sheep manure, sesame seed (*Sesamum indicum*), chia (*Salvia hispanica*) and black bean (*Phaseolus vulgaris*). Supplements were ground (2 mm screen), autoclaved in high-density polyethylene bags at 1.05 kg/cm^2^ (121 °C) for 20 min and mixed into the substrate both at spawning and prior to casing (9% dry wt each time). Two trials of 13 and 16 treatments were conducted using single or combined supplements (Tables 1, 2).

**Table 1:** Treatments used in the first supplementation trial (four ingredients) for the cultivation of *A. bisporus* on self-heated pasteurized Pangola grass

Treatment	Supplement			
	Soybean	Sheep manure	Wheat bran	Sesame seed
1	0.125	0.125	0.625	0.125
2	0	1	0	0
3	0.333	0	0.333	0.333
4	0.333	0.333	0.333	0
5	0	0	0	1
6	0.25	0.25	0.25	0.25
7	0.125	0.125	0.125	0.625
8	0.125	0.625	0.125	0.125
9	0.625	0.125	0.125	0.125
10	0.333	0.333	0	0.333
11	1	0	0	0
12	0	0	1	0
13	0	0.333	0.333	0.333

**Table 2:** Treatments used in the second supplementation trial (five ingredients) for the cultivation of *A. bisporus* on self-heated pasteurized Pangola grass

Treatment	Supplement				
	Soybean	Sesame seed	Black beans	Wheat bran	Chia
1	0.1	0.6	0.1	0.1	0.1
2	0	1	0	0	0
3	0.1	0.1	0.1	0.1	0.6
4	0.25	0	0.25	0.25	0.25
5	0	0	0	1	0
6	0.25	0.25	0	0.25	0.25
7	0.25	0.25	0.25	0	0.25
8	0.1	0.1	0.6	0.1	0.1
9	0.6	0.1	0.1	0.1	0.1
10	0	0	0	0	1
11	0	0.25	0.25	0.25	0.25
12	1	0	0	0	0
13	0	0	1	0	0
14	0.2	0.2	0.2	0.2	0.2
15	0.25	0.25	0.25	0.25	0
16	0.1	0.1	0.1	0.6	0.1

### Substrate preparation

Substrates were homogenized in a mixer MC-50 (Maquinaria Agropecuaria HML, Xalapa, Mex.), placed in a wooden crate measuring 1 m^3^, and then pasteurized by allowing the temperature to rise to 60–65 °C. After 30 h, it was removed, turned and placed back in the crate. The process was completed after 45 h by aerating the substrate to decrease the temperature (Sánchez et al. 2016; Morales and Sánchez 2017).

Two batches (20 kg each) of full Phase II mushroom compost, designated C_1_ and C_2_, were obtained from two mushroom companies located near Mexico City and Xalapa, Veracruz. The composts were prepared using the following ingredients: wheat straw, chicken manure, canola, urea an gypsum. Full Phase II mushroom compost two (C_2_) had the following composition: moisture content 69.2%, pH 7.6, Nitrogen 2.2%, C/N ratio 15–17, and ashes 25%.

### Cultivation

Substrate and spawn (5% inoculation rate, unless indicated differently) were mixed manually in 1 kg portions and placed in transparent polyethylene bags. To allow gas exchange, the upper end of the plastic bag was fitted into a plastic cylinder (4 cm diameter) and the hole covered with a clean white paper towel. Incubation lasted 3 weeks at 24–26 °C and after the substrate was colonized by the mushroom mycelium, the bag was opened and a casing (peat: lime: water 1:1:3; no thermal treatment) overlay (4 cm deep) was applied. The substrate was further incubated at 18 °C and 90% relative humidity for three more weeks. Irrigation was applied daily. When used, supplements were mixed into the substrate at spawning and before applying the casing (9% each time). Mushrooms were harvested (three breaks) when the pileus was open and the veil broken. No attempts were made to control weed molds and diseases.

### Treatments

To test the technical feasibility of *A. bisporus* cultivation on self-heated pasteurized substrates, three substrates without supplements were assayed initially. Two sets of supplementation treatments were then carried out, the first with four ingredients and 13 treatments, and the second with five ingredients and 16 treatments (Tables 1, 2). Supplements (on a dry weight basis) were added 9% at spawning and 9% at casing. Controls were two non-supplemented full phase composts (C_1_ and C_2_) using two spawning rates of 1 and 5% for C_1_, and only 5% for C_2,_ as indicated.

### Chemical analysis

To determine Carbon/Nitrogen (C/N) ratios, reducing sugar and lipid levels, 200 g samples were dried in a 65 °C oven for 5 days, ground into a fine powder and sent to the Bromatology Laboratory (Ecosur) for analysis. Carbon and nitrogen levels were determined using a Flash 2000 Analyzer (Thermo Fischer Scientific, http://www.thermoscientific.com/). Reducing sugars were measured using the 3,5 dinitrosalicylic acid (DNS) method (Miller 1959), and total lipids were determined according to Williams (1984).

### Evaluated parameters

Biological efficiency (BE) values were computed using the following calculation: fresh mushroom weight divided by dry substrate weight and multiplied by 100. The production rate (PR) was calculated by dividing BE by the number of days needed to obtain three flushes. Mean size of mushrooms (MMS) was calculated by dividing the weight of mushrooms by the number of mushrooms harvested per bag. Yields were estimated by dividing the production of each bag by the horizontal area exposed to mushroom production and are expressed in kg/m^2^. The incidence of contamination was estimated visually: at the end of the spawn run, and after the first harvest, the percentage of contaminated area per each substrate bag was estimated. The result was expressed as an average.

### Statistical analysis

A completely randomized design with five repetitions was used (Tables 3, 4, 5). To investigate the impact of the ingredients of the supplement mixtures on yield, a mixture design was used (Table 7). In each case, five repetitions were used and an analysis of variance and a mean separation was evaluated using Tukey’s test with a significance threshold of p < 0.05. Statistical analysis was carried out using JMP version 4 (SAS 2000).

**Table 3:** Production variables of *Agaricus bisporus* cultivated on three substrates pasteurized by self-heating

Substrate	Production (g/bag)	BE (%)	MMS (g)	PR (%)	Y (kg/m^2^)
Mixture	132.0 ± 56.0^a^	37.7 ± 16.0^a^	36.6 ± 5.1^a^	1.2 ± 0.5^a^	5.5 ± 2.3^a^
Corncob	65.0 ± 41.1^b^	18.5 ± 11.7^b^	35.0 ± 26.1^a^	0.6 ± 0.3^b^	2.7 ± 1.7^b^
Grass	181.2 ± 55.7^a^	51.7 ± 15.9^a^	31.25 ± 9.7^a^	1.7 ± 0.5^a^	7.6 ± 2.3^a^
p	0.000118	0.000118	0.9367	0.000118	0.000118

Same letter in the same column indicates no statistical difference between substrates (alpha = 0.05)

*BE* biological efficiency, *MMS* mean mushroom size (g), *PR* production rate (%), *Y* yield

**Table 4:** Production variables of *A. bisporus* cultivated on Pangola grass supplemented (9%) with 13 different mixtures at spawning and casing

Treatment	Supplement				Production variables				
	Soy bean	Sheep manure	Wheat bran	Sesame seed	Production (g/1 kg wet substrate)	BE (%)	MMS (g)	PR (%)	Y (kg/m^2^)
C_1_1%	–	–	–	–	531.2 ± 202.6^ab^	151.7 ± 57.9^ab^	54.2 ± 20.6^b^	5.0 ± 1.9^ab^	22.4 ± 8.5^ab^
C_1_5%	–	–	–	–	623.5 ± 56.7^a^	178.1 ± 16.2^a^	43.1 ± 10.8^b^	5.9 ± 0.5^a^	26.3 ± 2.3^a^
0	–	–	–	–	240.6 ± 58.6^d^	68.7 ± 16.7^d^	45.2 ± 12.0^b^	2.2 ± 0.5^d^	10.1 ± 2.4^d^
1	0.125	0.125	0.625	0.125	540.2 ± 106.8^ab^	154 ± 30^ab^	50.0 ± 12.4^b^	5.1 ± 1.0^ab^	22.7 ± 4.5^ab^
2	0	1	0	0	393.2 ± 151.8^bcd^	112.3 ± 43.3^bcd^	41.4 ± 9.8^b^	3.7 ± 1.4^bcd^	16.6 ± 6.4^bcd^
3	0.333	0	0.333	0.333	497.6 ± 47.9^abc^	142.1 ± 13.7^ab^	39.8 ± 11.1^b^	4.7 ± 0.4^ab^	20.9 ± 2.0^abc^
4	0.333	0.333	0.333	0	433.4 ± 57.2^abc^	123.8 ± 16.3^abc^	39.2 ± 7.0^b^	4.1 ± 0.5^abc^	18.2 ± 2.4^abc^
5	0	0	0	1	363.2 ± 31.8^bcd^	103.7 ± 9.0^bcd^	44.2 ± 7.1^b^	3.4 ± 0.3^bcd^	15.3 ± 1.3^bcd^
6	0.25	0.25	0.25	0.25	493.2 ± 57.0^abc^	140.9 16.3^abc^	50.0 ± 8.7^b^	4.6 ± 0.5^ab^	20.8 ± 2.4^ab^
7	0.125	0.125	0.125	0.625	401.6 ± 30.0^bcd^	114.7 ± 8.5^bcd^	42.8 ± 11.6^b^	3.8 ± 0.2^bcd^	16.9 ± 1.2^bcd^
8	0.125	0.625	0.125	0.125	384.4 ± 42.3^bcd^	109.8 ± 12.1^bcd^	48.0 ± 7.6^b^	3.6 ± 0.4^bcd^	12.2 ± 1.7^bcd^
9	0.625	0.125	0.125	0.125	391.6 ± 118^bcd^	111.8 ± 33.7^bcd^	46.0 ± 13.9^b^	3.7 ± 1.1^bcd^	16.5 ± 1.7^bcd^
10	0.333	0.333	0	0.333	394.6 ± 89.5^bcd^	112.7 ± 25.8^bcd^	35.2 ± 11.1^b^	3.7 ± 0.8^bcd^	16.6 ± 3.7^bcd^
11	1	0	0	0	273.2 ± 52.6 ^cd^	78.1 ± 15.03 ^cd^	41.5 ± 8.0^b^	2.6 ± 0.5 ^cd^	11.5 ± 2.2 ^cd^
12	0	0	1	0	323.2 ± 52.6^bcd^	92.1 ± 47.5^bcd^	41.0 ± 29.34^b^	3.07 ± 1.5^bcd^	13.6 ± 7.0^bcd^
13	0	0.333	0.333	0.333	407.6 ± 235.1^abc^	116.4 ± 67.1^abc^	77.2 ± 52.1^a^	3.8 ± 2.2^abc^	17.1 ± 9.9^abc^
p	–	–	–	–	0.0007	3.31e-07	4.524e-05	3.31e-07	3.31e-07

Same letters in the same column indicate no statistical difference between treatments (alpha = 0.05)

*BE* biological efficiency, *MMS* mean mushroom weight (g), *PR* production rate (%), *Y* yield

**Table 5:** Production variables of *A. bisporus* cultivated on Pangola grass and supplemented (9% dw) at spawning and casing with different mixtures of soybean, sesame seed, black bean, wheat bran and chia

Treatment	Supplement					Production variables				
	Soy bean	Sesame seed	Black beans	Wheat bran	Chía	Production (g/1 kg wet substrate)	BE (%)	MMS (g)	PR (%)	Y (kg/m^2^)
C_2_5%	–	–	–	–	–	438.4 ± 105.8^bc^	125.3 ± 30.2^bc^	53.4 ± 12.8^abc^	4.1 ± 1.0^bc^	18.4 ± 4.4^bc^
0	–	–	–	–	–	155 ± 59.9^d^	44.2 ± 17.1^d^	38.0 ± 6.7^bc^	1.4 ± 0.5^d^	6.5 ± 2.5^d^
1	0.1	0.6	0.1	0.1	0.1	598 ± 90.0^ab^	170.9 ± 25.7^ab^	47.6 ± 12.7^ab^	5.6 ± 0.8^ab^	25.2 ± 2.5^ab^
2	0	1	0	0	0	450 ± 112.2^abc^	128.6 ± 32.0^abc^	43.4 ± 5.0^ab^	4.2 ± 1.2^abc^	18.9 ± 4.7^abc^
3	0.1	0.1	0.1	0.1	0.6	543.6 ± 138.1^abc^	155.3 ± 39.4^abc^	36.4 ± 8.1^c^	5.1 ± 1.3^abc^	22.9 ± 5.8^abc^
4	0.25	0	0.25	0.25	0.25	617.2 ± 128.7^a^	176.3 ± 36.7^a^	48.0 ± 10.8^abc^	5.8 ± 1.3^a^	26.0 ± 5.4^a^
5	0	0	0	1	0	538.4 ± 128.4^abc^	153.8 ± 27.1^abc^	44.0 ± 9.4^abc^	5.1 ± 1.2^abc^	22.7 ± 5.4^abc^
6	0.25	0.25	0	0.25	0.25	449.2 ± 95.0^abc^	128.3 ± 22.9^abc^	58.2 ± 9.2^abc^	4.2 ± 0.9^abc^	18.9 ± 4.0^abc^
7	0.25	0.25	0.25	0	0.25	475.6 ± 80.3^abc^	135.9 ± 24.0^abc^	43.0 ± 11.8^abc^	4.5 ± 0.8^abc^	20.0 ± 3.3^abc^
8	0.1	0.1	0.6	0.1	0.1	471.8 ± 84.1^abc^	134.8 ± 24.0^abc^	41.6 ± 11.0^abc^	4.4 ± 0.7^abc^	19.9 ± 3.5^abc^
9	0.6	0.1	0.1	0.1	0.1	423 ± 10.36^bc^	120.9 ± 2.9^bc^	53.0 ± 5.7^abc^	4.0 ± 0.8^bc^	17.8 ± 0.4^bc^
10	0	0	0	0	1	494.8 ± 88.7^abc^	141.4 ± 15.0^abc^	66.8 ± 5.7^a^	4.7 ± 0.8^abc^	20.8 ± 3.7^abc^
11	0	0.25	0.25	0.25	0.25	369 ± 52.7^c^	105.4 ± 15.0^c^	43.8 ± 3.0^abc^	3.5 ± 0.5^c^	15.5 ± 2.2^c^
12	1	0	0	0	0	370.2 ± 41.4^c^	105.8 ± 11.8^c^	51.8 ± 9.4^abc^	3.5 ± 0.3^c^	15.6 ± 1.7^c^
13	0	0	1	0	0	442.4 ± 53.9^abc^	126.4 ± 15.4^abc^	64.0 ± 31.3^ab^	4.2 ± 0.5^abc^	18.6 ± 2.7^abc^
14	0.2	0.2	0.2	0.2	0.2	451 ± 38.3^abc^	128.9 ± 10.9^abc^	45.4 ± 11.1^abc^	4.2 ± 0.3^abc^	19.0 ± 1.6^abc^
15	0.25	0.25	0.25	0.25	0	448.2 ± 34.3^abc^	128.1 ± 9.8^abc^	45.0 ± 4.4^abc^	4.2 ± 0.3^abc^	18.9 ± 1.4^abc^
16	0.1	0.1	0.1	0.6	0.1	451.2 ± 36.9^abc^	128.9 ± 10.5^abc^	48.8 ± 8.3^abc^	4.2 ± 0.3^abc^	19.0 ± 1.5^abc^
p	–	–	–	–	–	1.958e-09	1.958e-09	0.002284	1.958e-09	1.958e-09

Same letters in the same column indicate no statistical difference between treatments (alpha = 0.05)

*BE* biological efficiency, *MMS* mean mushroom weight (g), *PR* production rate (%), *Y* yield

## Results

### Cultivation on substrates without supplements

Table 3 shows the production variables of *A. bisporus* on three substrates pasteurized by self-heating. Biological efficiency values varied between 18.5 (corncob) and 51.7% (Pangola grass), Production Rates between 0.6 and 1.7%, and yields between 2.7 and 7.6 kg/m^2^. Statistical analysis indicated that the cultivation of the mushroom on the mixture or on Pangola grass alone gave similar results, and that both substrates were significantly different from corncob, whose results were lower (p = 0.000). The weight of individual mushrooms ranged between 31.2 and 36.6 grams, and no significant difference was found between all three substrates (p = 0.9367).

### Cultivation on supplemented substrate

Table 4 shows the production variables of *A. bisporus* cultivated on Pangola grass supplemented with 13 different mixtures of soybean, sheep manure, wheat bran and sesame seed. Biological Efficiency values varied from 68.7% (T_0_, non supplemented Pangola grass) to 178.1% (C_2_, full compost with 5% spawning rate). Statistical analysis indicated significant differences among treatments (p = 0.000). Table 5 shows the production variables of *A. bisporus* in a second evaluation trial, cultivated on Pangola grass supplemented with 16 different mixtures of soybean, sesame seed, black bean, wheat bran and chia. Biological Efficiency values varied from 44.2 ± 17.1 (T_0_) to 176.3 ± 36.7 (T_4_, formulation with 25% each of four ingredients excluding sesame seed). Statistical analysis indicated significant differences among treatments, establishing four statistical groups. Group “a” with the highest BE values varying between 128.1 and 176.3%. T_4_ generated a BE value significantly higher than that obtained with control Phase II compost (125.3%; p = 0.000), and also produced larger mushrooms (p = 0.0022) and higher yields (p = 0.000) than the full compost. Yields varied between 6.5 kg/m^2^ (T_0_) and 26.0 kg/m^2^ (T_4_).

### Effect of supplements

Table 6 shows the nutritional value of the substrates and supplements used in these experiments. Since the substrates used for cultivating *A. bisporus* were of low nutritional value, BE and yield values recorded with the substrates alone were also low. Therefore, in order to improve these production parameters, it was necessary to create better formulations through the application of supplements. In all cases, the supplemented treatments produced higher yields than the control (Pangola grass alone, T_0_) and the highest BE values recorded were comparable to (Table 4, p = 0.000), or even better than (Table 5, p = 0.000), those obtained with the full Phase II compost. A correlation analysis was performed between supplement content (nitrogen, C/N, lipids and reducing sugar) and production variables (BE, MMS, PR and yield). Correlation coefficients varied between −0.54 and 0.36 (data not shown), indicating there was no correlation between the variables tested.

**Table 6:** Chemical composition of substrates and supplements used in this investigation

Substrates	Carbon (g/100 g)	Nitrogen (g/100 g)	C/N	Fat (g/100 g)	Reducing sugars (g/100 g)
Corncob	51.92	0.42	123.6	–	34.92
Grass	49.24	0.82	60.0	1.3	17.2
Mixture	51.12	0.56	91.2	–	16.11
Supplements					
Sesame seed	50.68	3.29	15.4	47.97	13.7
Soybean	42.46	5.61	7.6	22.52	30.1
Sheep manure	38.2	2.24	17.1	1.0512	15.3
Wheat bran	35.64	2.57	13.9	4.53	66.8
Chia	47.84	3.37	14.2	33.49	18.65
Black beans	35.99	2.95	12.2	1.65	85.14

Analyses of supplements using a mixture design (Table 7) indicated that the principal components of the substrate exerted a significant effect (p values between 0.000 and 0.001) but there were no significant differences among the interactions between those components (p values >0.05).

**Table 7:** Parameter estimates of the mixture design of two supplementation trials evaluated

Term		Estimate	Std error	t ratio	Prob> |t|
Trial 1					
Intercept	Zeroed	0	0	–	–
Soybean (mixture) & RS		75.828024	12.3874	6.12	0.0017
Sheep manure (mixture) & RS		110.41891	12.3874	8.91	0.0003
Wheat bran (mixture) & RS		97.727195	12.3874	7.89	0.0005
Sesame seed (mixture) & RS		101.22084	12.3874	8.17	0.0004
Soybean (mixture)*sheep manure (mixture)	Biased	−139.0873	112.6716	−1.23	0.2719
Soybean (mixture)*wheat bran (mixture)	Biased	285.2221	112.6716	2.53	0.0524
Sheep manure (mixture)*wheat bran (mixture)	Biased	181.01433	121.169	1.49	0.1954
Soybean (mixture)*sesame seed (mixture)	Biased	224.53256	121.169	1.85	0.1231
Sheep manure (mixture)*sesame seed (mixture)	Zeroed	0	0	–	–
Wheat bran (mixture)*sesame seed (mixture)	Zeroed	0	0	–	–
Trial 2					
Intercept	Zeroed	0	0	–	–
Soybean (mixture) & RS	100.61448	24.08483	4.18	0.0058	
Sesame seed (mixture) & RS	140.73471	24.08483	5.84	0.0011	
Black beans (mixture) & RS	125.73009	24.08483	5.22	0.002	
Wheat bran (mixture) & RS	151.73587	24.08483	6.3	0.0007	
Chía (mixture) & RS	143.47691	24.08483	5.96	0.001	
Soybean (mixture)*sesame (mixture)	63.101291	384.2923	0.16	0.875	0.891
Soybean (mixture)*black bean (mixture)	344.78915	384.2923	0.9	0.4042	0.1963
Sesame seed (mixture)*black beans (mixture)	−242.7836	382.7513	−0.63	0.5493	0.3501
Soybean (mixture)*wheat bran (mixture)	−183.1043	343.033	−0.53	0.6127	0.8353
Sesame seed (mixture)*wheat bran (mixture)	Zeroed	0	0	–	–
Black bean (mixture)*wheat bran (mixture)	Zeroed	0	0	–	–
Soybean (mixture)*chía (mixture)	Biased	151.41592	343.033	0.44	0.6744
Sesame seed (mixture)*chía (mixture)	Zeroed	0	0	–	–
Black bean (mixture)*chía (mixture)	Zeroed	0	0	–	–
Wheat bran (mixture)*chía (mixture)	Zeroed	0	0	–	–

### Contamination

Most of the spawned bags (replicates) produced mushrooms, although some exhibited poor yields due to the presence of contaminants: when the mushroom was cultivated on corncob, *Trichoderma* sp. was present in three of 20 bags spawned with *A. bisporus* (15%). Also, after the second flush on corncob, the myxomycete, *Didymium iridis,* was detected. To our knowledge, this is the first time this myxomycete has been reported to be a contaminant of mushroom substrate. Among the 80 bags spawned for the first supplemented trial on Pangola grass (Table 4), both treatments T_12_ and T_13_ had one bag contaminated with the mold *Trichoderma* sp. by the end of the colonization period. These two bags did not produce mushrooms. One bag in each of T_11_ and T_12_ were also about 20% contaminated with the same mold by the end of the first harvest, which would explain the low mushroom production recorded with these two supplements (soybean and wheat bran alone). The general incidence of contamination by *Trichoderma* sp was 6.6% in Supplementation Trial 1, and the fact that contamination appeared only in some bags of the last treatments suggests it occurred during spawning. For the second Supplementation trial (Table 5), also using Pangola grass as the substrate, none of the 90 bags spawned were contaminated and all the bags produced mushrooms on a regular basis.

## Discussion

The results obtained clearly demonstrate that it is possible to use the technique of pasteurization by self-heating to produce *A. bisporus* Portobello mushrooms. When the substrates were used alone, BE and yield values obtained were low compared to commercial data such as estimated average yields of button mushrooms in the USA (26.9 kg/m^2^ or 6.33 lb/sq foot, NASS 2016). These low yields may be due to the low nutritive value of the substrates used (nitrogen content between 0.42 and 0.82, Table 6). However, it is noteworthy that full substrate colonization was achieved in less than three weeks, suggesting a certain selectivity for mushroom growth. The selectivity observed on the traditional full mushroom compost for the growth of *A. bisporus* is based on the presence of thermophilic fungi developed during phase II pasteurization (Straatsma et al. 1989, 1994; Wiegant et al. 1992). However, in the case of self-heating pasteurization, the total length of the treatment and the thermophilic phase was less than 45 h, so the substrate was less degraded and thermophilic microorganisms had shorter time to colonize the raw substrate. The raw material was pasteurized scarcely in two days, a short time compared with the phase II composting method that requires 12–20 days (Zied et al. 2010).

In previous investigations, researchers have shown that Phase I is not a prerequisite for Phase II (Wiegant et al. 1992; Straatsma et al. 1994; Sánchez et al. 2008; Sánchez and Royse 2009; Coello-Castillo et al. 2009). Also, by using sterile substrates, it has been demonstrated that phase II is also not necessary to grow *A. bisporus* (San Antonio 1971; Sanchez and Royse 2001; Bechara et al. 2005). In this work, it was found that *A. bisporus* can be cultivated in several pasteurized substrate that have not received a phase I or Phase II process. The ability to colonize and then to fructify in a self pasteurized substrate could be linked to the ligninolytic capacity of the white button mushroom (Kabel et al. 2017; ten Have et al. 2003; Bonnen et al. 1994) but also to the microbiota associated to the mycosphere (Torres-Ruiz et al. 2016). Therefore, it would be interesting to undertake further studies on this system to understand better the microbial diversity and its relationship on the development of *A. bisporus.*

Sánchez et al. (2008) used pasteurized grass (6 h, 60 °C) for *A. bisporus* production and obtained 11% BE and 25 g mean mushroom weight. Differences may be due to strain difference, but also to a possible beneficial effect of the microbiota developed in the self-heated pasteurized substrate (Torres-Ruiz et al. 2016).

The data obtained through the supplementation trials (BE = 176.3%; Y = 26 kg/m^2^) are interesting since Schisler (1982), for example, stated that BE values between 80 and 90% are already attractive commercially. They also compare favourably with previously results (BE = 73.1% and Y = 12.7 kg/m^2^) obtained by cultivating brown varieties of *A. bisporus* on non-conventional, pasteurized substrates (Coello-Castillo et al. 2009; Sánchez et al. 2008). Furthermore, they exceed BE and yield values (99.5–108.2% and 25.8–28.6 kg/m^2^, respectively) obtained by Pardo Giménez et al. (2016) who cultivated a white hybrid of *A. bisporus* on unsupplemented and supplemented (with defatted pistachio meal) Phase III compost. Sánchez and Royse (2009) using a *Scytalidium thermophilum*-colonized and supplemented substrate obtained BE and yield values of 99.3% and 21.19 kg/m^2^, respectively.

With regard to supplementation, our data with soybean, a supplement used successfully on a commercial scale since first reported by Schisler and Sinden (1962), confirmed those earlier results. Sheep manure, a supplement reported to be beneficial for *A. bisporus* cultivation (Mee 1978; Sanchez and Royse 2001), increased yields when used at low concentrations. The manure was obtained in dry form and no information about conditions of drying were available. In order to preserve quality, the drying process may need to be carefully controlled. Sesame seed is a grain high in fat and nitrogen. This product contributed to increased mushroom production only when it was applied at low concentrations in the supplementation formula. Conversely, chia, with a similar nitrogen content to sesame seed and less fat, had a positive impact on mushroom production. Comparing the treatments where ingredients were applied individually, chia and wheat bran showed higher increase in BE than the other ingredients (BE = 141.4 and 153.8%, respectively, Table 4). Specific components of each supplement with regard to amino acids, fatty acids, etc. may provide an explanation for why some mixtures promoted fruiting whereas others did not.

Supplementation is a highly complex variable where several group of formulations are recommended including proteins, lipids and carbohydrates (Wheeler and Wach 2006). It may be necessary to investigate micronutrients and individual ingredients present in the supplements in order to detect positive correlations. In this context, it has been reported that a single amino acid (isoleucine) positively stimulates mushroom yields when administered into the mushroom compost after the second flush (Royse and Sánchez 2008).

Successful colonization of the substrate by the mushroom and the low level of contamination recorded indicate that the use of raw substrates pasteurized by self-heating is a viable alternative method for cultivating Portobello mushrooms. The use of adequate supplement formulations improves mushroom development and allows the grower to obtain better yields statistically comparable to those obtained using traditional substrates (Phase II and III composts).

In regard to scaling up of the process, the self-heating pasteurization technique should be further studied (Sánchez et al. 2016). If conditions are improved, the duration of treatment may be reduced. Also, optimizing aeration would facilitate removal of the mass and reduce heat loss during turning. The technique used in this work is suitable for small growers because a crate of 1 m^3^ can process about 220 kg of grass or 380 kg corncobs with 65% moisture. If processing a larger amount of substrate is required, it would be possible to increase the volume of the container. However, the question rises to what extent it would be feasible, referring to the traditional technique of two phases. The larger the mass, the greater the heat generated, but also the greater the effort required to obtain a uniform temperature throughout the whole substrate, and also the greater the difficulties to moisten the substrate homogeneously.

## Abbreviations

C/N: carbon/nitrogen ratios
DNS: 3,5 dinitrosalicilic acid
BE: biological efficiency
PR: production rate
MMS: mean size of mushrooms
Y: yield

## References

[CR1] Avendaño-Hernandez RJ, Sánchez JE (2013). Self-pasteurised substrate for growing oyster mushrooms (*Pleurotus* spp.). Afr J Microbiol Res.

[CR2] Barrios-Espinoza BM, Moreno-Ruiz L, Sánchez-Vázquez JE (2009). Composteo en cajones de madera como pretratamiento del sustrato para cultivar *Pleurotus ostreatus*. Revista Mexicana De Micología.

[CR3] Bechara M, Heinemann P, Walker P, Romaine CP (2005). Cultivation of *Agaricus bisporus* on a mixture of cereal grain spawn and delayed-release nutrient supplement. Mush News.

[CR4] Bechara MA, Heinemann P, Walker PN, Romaine CP (2006). Non-composted grain-based substrates for mushroom production (*Agaricus bisporus*). Trans ASABE.

[CR5] Beyer DM (2017) Impact of the mushroom industry on the environment. Penn State Extension. http://extension.psu.edu/plants/vegetable-fruit/mushrooms/mushroom-substrate/impact-of-the-mushroom-industry-on-the-environment. Accessed 27 March 2017

[CR6] Bonnen AM, Anton LH, Orth AB (1994). Lignin-degrading enzymes of the commercial button mushroom *Agaricus bisporus*. Appl Env Microbiol.

[CR7] Coello-Castillo MM, Sánchez JE, Royse DJ (2009). Production of *Agaricus bisporus* on substrates pre-colonized by *Scytalidium thermophilum* and supplemented at casing with protein-rich supplements. Bioresour Technol.

[CR8] Durrant AJ, Wood DA, Cain RB (1991). Lignocellulose biodegradation by *Agaricus bisporus* during solid substrate fermentation. J Gen Microbiol.

[CR9] Gerrits JPG, Amsing JGM, Straatsma G, Van Griensven LJLD (1995). Phase I process in tunnels for the production of *Agaricus bisporus* compost with special reference to the importance of water. Mush Sci.

[CR10] Hernández D, Yamasaki K, Sánchez JE (2003). A simple procedure for preparing substrate for the production of *Pleurotus ostreatus*. Bioresour Technol.

[CR11] Kabel MA, Jurak E, Mäkela MR, de Vries RP (2017). Occurrence and function of enzymes for lignocellulose degradation in commercial *Agaricus bisporus* cultivation. Appl Microbiol Biotechnol.

[CR12] Laborde J, Lanzi G, Francescutti B, Giordani E, Chang ST, Buswell JA, Chiu SW (1993). Indoor composting: general principles and large scale development in Italy. Proceedings of the 1st international conference on mushroom biology and mushroom products.

[CR13] Mamiro DP, Royse DJ, Beelman RB (2007). Yield, size and mushroom solids content of *Agaricus bisporus* produced on non-composted and spent mushroom compost. World J Microbiol Biotechnol.

[CR14] Mee HM (1978) Mushroom composting. US patent 4, 127, 964

[CR15] Miller G (1959). Use of dinitrosalicylic acid reagent for determination of reducing sugar. Anal Chem.

[CR16] Miller FC, Harper ER, Macauley BJ, Gulliver A (1990). Composting based on moderately thermophilic and aerobic conditions for the production of commercial growing compost. Aust J Exp Agric.

[CR17] Morales V, Sánchez JE (2017) Self heating pasteurization of substrates for mushrooms cultivation. Int J Med Mush 19(5) **(in press)** 10.1615/IntJMedMushrooms.v19.i5.90 28845776

[CR18] NASS (2016) Mushrooms. National Agricultural Statistics Service (NASS). Agricultural Statistics Board, United States Department of Agriculture (USDA). http://usda.mannlib.cornell.edu/usda/current/Mush/Mush-08-19-2016.pdf. Accessed 27 March 2017

[CR19] Pardo-Giménez A, Catalán L, Carrasco J, Alvarez-Ortí M, Zied D, Pardo J (2016). Effect of supplementing crop substrate with defatted pistachio meal on *Agaricus bisporus* and *Pleurotus ostreatus* production. J Sci Food Agric.

[CR20] Royse DJ, Sánchez JE (2008). Supplementation of 2nd break mushroom compost with isoleucine, leucine, valine, phenylalanine, Fermenten^®^ and SoyPlus^®^. World J Microbiol Biotechnol.

[CR21] Royse DJ, Baars J, Tan Q, Zied DC (2016). Current overview of mushroom production in the world. Edible and medicinal mushrooms: technology and applications.

[CR22] San Antonio JP (1971). A laboratory method to obtain fruit from cased grain spawn of the cultivated mushroom, *Agaricus bisporus*. Mycologia.

[CR23] Sanchez JE, Royse DJ (2001). Adapting substrate formulas used for shiitake for production of brown *Agaricus bisporus*. Bioresour Technol.

[CR24] Sánchez JE, Royse DJ (2009). *Scytalidium thermophilum*-colonized grain, corncobs and chopped wheat straw substrates for the production of *Agaricus bisporus*. Bioresour Technol.

[CR25] Sánchez JE, Mejía L, Royse DJ (2008). Pangola grass colonized with *Scytalidium thermophilum* for the production of *Agaricus bisporus*. Bioresour Technol.

[CR26] Sánchez JE, Moreno L, Andrade-Gallegos R (2016) Low input technology for growing mushrooms: self-heating pasteurization. WSMBMP Bulletin 15: http://wsmbmp.org/Bol15/1.html. Accessed 3 March 2017

[CR27] SAS (2000) JMP 4.0, Statistical discovery software. SAS Institute. Campus Drive, Cary, NC 27513 USA. ISBN 1-58025-631-7

[CR28] Schisler LC (1982) Biochemical and mycological aspects of mushroom composting. In: Penn State handbook for commercial mushroom growers Special publication. College of Agricultural Sciences, The Pennsylvania State University, State College, p 3–10

[CR29] Schisler LC, Sinden J (1962). Nutrient supplementation of mushrooms compost at spawning. Mush Sci.

[CR30] Sinden JW, Hauser E (1950). The short method of composting. Mush Sci.

[CR31] Straatsma G, Gerrits JPG, Augustijn MPAM, Op den Camp HJM, Vogels GD, Van Griensven LJLD (1989). Population dynamics of *Scytalidium thermophilum* in mushroom compost and stimulatory effect on growth rate and yield of *Agaricus bisporus*. J Gen Microbiol.

[CR32] Straatsma G, Olijnsma TW, Gerrits JPG, Amsing JGM, Op den Camp JJM, Van Griensven LJLD (1994). Inoculation of *Scytalidium thermophilum* in button mushroom compost and its effect on yield. Appl Environ Microbiol.

[CR33] Straatsma G, Samson RA, Olijnsma TW, Gerrits JPG, Op den Camp HJM, Van Griensven LJLD (1995). Bioconversion of cereal straw into mushroom compost. Can J Bot.

[CR34] ten Have R, Wijngaard H, Aries-Kronenburg NAE, Straatsma G, Schaap PJ (2003). Lignin degradation by *Agaricus bisporus* accounts for a 30 increase in bioavailable holocellulose during cultivation on compost. J Agric Food Chem.

[CR35] Till O (1962). Cultivation of mushrooms on sterile substrate and reutilization of spent compost. Mush Sci.

[CR36] Torres-Ruiz E, Sánchez JE, Guillén-Navarro GK, Ramos-Pérez DG, Royse DJ (2016). Microbial promoters of mycelial growth, fruiting and production of *Pleurotus ostreatus*. Sydowia.

[CR37] Villa-Cruz V, Huerta G, Sánchez JE (1999). Solid fermentation of a corn cob-coffee pulp mixture for the cultivation of *Pleurotus ostreatus*. Micol Neotrop Apl.

[CR38] Wheeler D, Wach MP (2006). Re-evaluating supplementation. Mushroom News.

[CR39] Wiegant WM, Wery J, Buitenhuis ET, De Bont JAM (1992). Growth promoting effect of thermophilic fungi on the edible mushroom *Agaricus bisporus*. Appl Environ Microbiol.

[CR40] Williams S (1984). Crude fat or ether extract. Official methods of analysis of the Association of Analytical Chemists.

[CR41] Wood DA, Leatham GF (1983). Lignocellulose degradation during the life cycle of *Agaricus bisporus*. FEMS Microbiol Lett.

[CR42] Wuest PJ (1982). Pasteurization during the mushroom grow cycle Penn State handbook for commercial mushroom growers.

[CR43] Zied DC, Almeida-Minhoni MT, Pardo González JE, Pardo Gimenez A (2010). A study of compost added to a casing technique in *Agaricus bisporus* cultivation from phase III bulk compost. HortScience.

